# Editorial: Targeting Membrane Proteins: Structure-Function-Dynamics Relationships

**DOI:** 10.3389/fchem.2022.862802

**Published:** 2022-03-03

**Authors:** Claire Colas, Ivet Bahar, Lei Shi, Josep Font Sadurni

**Affiliations:** ^1^ Department of Pharmaceutical Sciences, University of Vienna, Vienna, Austria; ^2^ Department of Computational and Systems Biology, School of Medicine, University of Pittsburgh, Pittsburgh, PA, United States; ^3^ Computational Chemistry and Molecular Biophysics Section, National Institute on Drug Abuse – Intramural Research Program, National Institutes of Health, Baltimore, MD, United States; ^4^ Transporter Biology Group, School of Medical Sciences, Faculty of Medicine and Health, University of Sydney, Sydney, NSW, Australia

**Keywords:** membrane proteins, drug targets, structure, function, dynamics

Recent years have seen significant advances in the structure-based characterization of the mechanisms of function of membrane proteins, in particular transporters, and in the development of allosteric modulator and pharmacological agents that regulate their function. This issue provides examples from recent progress in the field, along with additional studies on ion channeling and secretion of proteins at the ER.

Solute carrier (SLC) transporters constitute a major membrane protein target class. The SLC6 family is one of the most studied SLC family, notably the monoamine transporter subgroup, containing neurotransmitter sodium symporters (NSS) such as norepinephrine, dopamine and serotonin transporters. The pharmacology of these transporters has been studied for many years due to their high therapeutic impact. Recently, particular emphasis has been put on structure-based methods, with the aim of deciphering the molecular determinants involved in ligand binding specificities. Three studies in this collection focus on SLC6 members. Frangos et al. analyze three allosteric sites described in literature for members of the SLC6 family, and explore how these sites can be targeted for the modulation of the glycine transporter GlyT2, an emerging target in the SLC6 family. Kickinger et al. on the other hand, describe the structural determinants for the activity of a novel class of inhibitors of the Betaine/GABA transporter BGT1 (a member of the GABA transporter subgroup of the SLC6 family). Romanazzi et al. draw attention to the role of bile acids, molecules derived from cholesterol, and in particular that of the obeticholic acid in regulating channel-like activity of dopamine transporter. Together, these studies provide new building blocks to further functionally characterize and modulate the activities of the members of this important family of neurotransmitter transporters, which could open new avenues for pharmacological treatments of SLC6-related brain disorders.

The current collection also has two interesting studies on antiporters. Antiporters are secondary transporters that transport two (or more) substrates, ions or small molecules, in opposite directions across the membrane. The first study is on a human zinc transporter 1 (hZnT1) that shows Zn^2+^/H^+^ antiporter activity in the presence of detergent and cholesteryl hemisuccinate. hZnT1 export Zn^2+^ from the cytoplasm to protect cells from Zn^2+^ toxicity. Cotrim et al. were able to express the soluble C-terminal domain of hZnT1 (hZnT1-CTD) in a bacterial expression system, and found an increase in the melting temperature of the hZnT1-CTD at acidic pH, while the small-angle X-ray scattering analysis indicated that hZnT1-CTD forms a dimer in solution with a V-shaped core. These findings provide a basis for the structure-function studies of hZnT1 and its close homologs. The second is on the archaeal Na^+^/Ca^2+^ exchanger, as a model system to study the mechanism of function of the superfamily of Ca^2+^/Cation antiporters. Khananshvili presents an extensive review of the current understanding of the mechanism of function of this exchanger.

Inwardly rectifying potassium (Kir) channels are regulated by ligands such as Na+ and H+, adenosine nucleotides, and lipid phosphatidyl-inositol 4,5-bisphosphate (PIP_2_). The latter is an essential activator for eukaryotic members of the Kir family, but its specific binding and action on Kir6.2 has eluded characterization despite the resolution of several X-ray and cryo-EM structures for PIP_2_-bound Kir channels. Bründl et al. simulated PIP_2_-induced gating events allowing for the channeling of potassium across the Kir6.2 pore of ATP-sensitive potassium channels, using the structural data available for PIP_2_-bound homologs Kir2 and Kir3 together with functional data. The study provides information on the molecular structure and dynamics of Kir pore in the presence of PIP_2_, as well as the order of events that enable the stepwise regulation of Kir6.2 activity by PIP_2_. Furthermore, the authors examined the effect of the point mutation L164P associated with Permanent Neonatal Diabetes, to show how this mutation influences pore geometry and disrupts stability.

The carboxy terminus of an endoplasmic reticulum (ER) resident proteins typically have an ER retention/retrieval sequence (ERS), which usually contains the canonical Lys-Asp-Glu-Leu (KDEL) motif. KDEL receptors (KDELRs) in the Golgi recognize the ERS and return the protein to the ER lumen. By combining machine learning-based modeling and experimental validation, Trychta et al. identified the ERS divergent from the canonical “KDEL” motif. Using molecular modeling and simulations, they showed that two representative non-canonical ERS can stably bind to the KDELRs, which employs interactions beyond the final four residues of the ERS. Their work established an integrated platform to predict whether a carboxy-terminal sequence acts as a putative ERS. The ability to predict which proteins may be secreted may shed light to the complex relationship between disruptions in ER homeostasis and diverse pathologies.

